# Medical Organization in Texas

**Published:** 1894-11

**Authors:** P. C. Coleman

**Affiliations:** Colorado, Texas


### Correspondence.

### Medical Organization in Texas.

COLORADO, TEXAS, October 18, 1894.

*Editor Texas Medical Journal:*

Certainly every reputable and ethical physician in Texas should be with you in the desire to see the State Association "absorb all the eligible material in the State, and become a power," but when we review the history of medical organization in the older States, all of them, compared with Texas, enjoying better facilities for gathering in State societies the profession, we must conclude we have no ground for indulging the hope such a desire will ever be realized.

As you suggest in your editorial in the September number of the JOURNAL, "the State is too large and the doctors live too far apart to ever hold them together in an *Association* by *Membership*."

This same objection applies to any and all plans, by "districts" or otherwise. You would have about your same number in attendance at annual meetings, and Dr. Larendon would receipt about the same number for annual dues, as under the present plan of organization.

The treasurer's books will show a large majority only pay their dues while in attendance on meetings of the Association. If such be the fact, one dollar a year per member would not pay expenses.

I do not believe a reduction in dues from five dollars to less will add any to the Association, for annual dues constitutes a small part of the expenses. Railroad fare, in many instances hundreds of miles, hotel bills, loss by absence from home, go to make up the larger part of the expenses, and these will remain.

With you, I regret we have so small a number of the physicians of the State in the Association, but I submit Texas is not lagging behind in medical organization. Let us see: California: Number of physicians, 2,700; number who belong to State society, 360, 13.33 per cent.; number who attend annual meetings, ——.

Alabama: Number physicians, 1,800; number who are members of the State society, 1,100, 61.11 per cent.; number who attend annual meetings, 225, 20.45 per cent.

Nebraska: Number of physicians, 1,100; number who are members of the State society, 350, 31.81 per cent.; number who attend annual meetings, 150, 43 per cent.

Mississippi: Number of physicians, 2,000; number who are members of the State society, 450, 22.50 per cent.; number who attend annual meetings, 150, 33 per cent. Mississippi has two State societies, and both are included in above figures.

Georgia: Number of physicians, 3,000; number who are members of the State society, 450, 15 per cent.; number who attend annual meetings, 175, 38.88 per cent.

Texas: Number of physicians, 4,000; number who belong to the State society, 386, 9.65 per cent.; number who attend annual meetings, 175, 45.33 per cent.

With reference to district, county, and medical organizations in cities, after observation, rendering me to some extent familiar with similar organizations in other States, I do not think Texas will suffer by comparison.

Again, Texas is not behind in her membership in the American Medical Association. Virginia, with over 2,800 physicians, has 38 members. North Carolina, 1,600 physicians, has 17 members. Mississippi has 2,000 physicians, 21 members. Alabama, 1,800 physicians, 20 members. Nebraska has 1,100 physicians, 68 members. Texas has 4,000 physicians, 71 members.

While it is true we have a small per cent. of the physicians in the State in the Association, compared with other States, and this is not as it should be, and is to be very much regretted, still we have a larger per cent. of our membership who attend annual meetings than others, as shown by above figures, and right here lies the secret of success, or failure. It is not in numbers alone, but how many can be induced to attend the annual meetings regularly.

I am under obligations to the secretaries of the State societies included in this letter, for their courtesy in giving information from their respective societies. It was my intention to include others, but it seems they did not deem my letters of sufficient importance to reply to them.

Very truly,

P. C. COLEMAN, M. D.

